# Current status of vitamin D_2_ deficiency among children in a region of China

**DOI:** 10.3389/fped.2024.1333769

**Published:** 2024-01-26

**Authors:** Jia Liu, Zhihua An, Na An, Yile Zhao, Guying Zhang, Deyun Zhao

**Affiliations:** Department of Pharmacy, Children’s Hospital of Hebei Province, Shijiazhuang, Hebei, China

**Keywords:** vitamin D, vitamin D_2_, clinical diseases, investigation, deficiency

## Abstract

**Background:**

The aim of this study was to explore the current status of vitamin D_2_ (VD_2_) deficiency in hospitalized children in a region of China.

**Methods:**

The instances of detection of vitamin D (VD) and VD_2_ in children who visited the hospital from January 2022 to May 2023 were analyzed retrospectively. Additionally, the relationships between VD_2_ level and gender and age were further analyzed. Furthermore, for departments with a high frequency of VD detection, the VD_2_ deficiencies in children with different diseases were further analyzed.

**Results:**

Among the different age groups, children aged 11–15 years exhibited the most severe VD_2_ deficiency, followed by those aged 7–10 years, 0–1 years, and 2–6 years. Moreover, 25(OH)D_2_ levels were significantly lower in children aged 7–10 years and 11–15 years compared with 2–6 years. Gender did not have an impact on the level of 25(OH)D_2_. When analyzing the orthopedics, dermatology, thoracic surgery, and nephroimmunology departments’ data on children's levels of 25(OH)D_2_, it was found that an average of approximately 76.56% had levels below <1.5 ng/ml compared to individuals with levels between >15 ng/ml and 100 ng/ml. The average ratio between individuals with <1.5 ng/ml vs. those with <15 ng/ml was found to be 91.22%.

**Conclusions:**

Children who came to the hospital were severely deficient in VD_2_. The degree of deficiency was related to age, but there was no gender difference. The phenomenon of VD_2_ deficiency was reflected in children with both skeletal and non-skeletal diseases.

## Introduction

Vitamin D (VD) is a steroid hormone that plays a crucial role in the growth and development of children. Within the VD family, the major members are ergocalciferol (vitamin D_2,_ VD_2_) and cholecalciferol (vitamin D_3_, VD_3_). VD_2_ primarily exists in fungi and some plants, while VD_3_ is synthesized from 7-dehydrocholesterol in human or animal skin through ultraviolet light photochemical action, serving as the primary source of vitamin D in the human body. Upon entering the liver, both VD_2_ and VD_3_ undergo conversion to 25-hydroxyvitamin D [25(OH)D] by 25-hydroxylase within hepatocyte microsomes. Subsequently, hydroxylation of 25(OH)D leads to its active form known as 1,25-dihydroxyvitamin D [1,25-(OH)_2_-D], which exerts physiological effects. VD has many metabolites, but there are no recognized metabolites for measuring the content of VD currently. The serum concentration of 25(OH)D is used as an indicator for assessing the level of VD due to a longer half-life (1). VD deficiency not only leads to skeletal diseases but also affects the occurrence and progression of various non-skeletal health outcomes, including autism, systemic lupus erythematosus, kidney disease, cardiovascular disease, immune system disorders, metabolic disorders, and nervous system disorders (2). Individuals with VD deficiency are more susceptible to these diseases compared to those with normal VD levels. In recent years, there have been many studies about VD and VD_3_, but research on VD_2_ is relatively insufficient. This study aimed to analyze the current status of VD_2_ deficiency in children visiting a hospital and lay the foundation for future research on VD_2_.

## Methods

### Subjects

Experienced doctors would empirically assess the possibility of VD deficiency in children based on their daily VD supplementation status and the presence of clinical symptoms such as constipation, sleep disorders, and calcium deficiency and arrange for VD testing. The study was conducted by a retrospective analysis of 11,506 children who underwent VD and VD_2_ testing in our hospital from January 2022 to May 2023, including 5,923 boys and 5,583 girls, with an average age of (4.82 ± 4.29) years. The children were divided into four groups according to age: 0–1 years, 2–6 years, 7–10 years, and 11–15 years.

### Definition of the normal range of VD

According to the standards of the clinical practice guidelines of the Endocrine Society of America and the expert consensus on the clinical application of VD in China (3), the nutritional status of children with VD was defined as follows: The normal range of 25(OH)D is 15 ng/ml to 100 ng/ml. The 25(OH)D level can reflect the normal VD nutritional status of children. The normal range of 25(OH)D_2_ is 1.5 ng/ml to 30 ng/ml. The 25(OH)D_2_ level indicates the VD_2_ nutritional status of children.

### Detection method of VD

Laboratory tests for VD were performed by personnel from the pharmacological laboratory of the Department of Pharmacy. Researchers collected 2 ml venous blood from children, centrifuged for 6 h to obtain serum, and stored frozen in a refrigerator at −20°C for subsequent test. During the detection process, 25(OH)D and 25(OH)D_2_ were detected by liquid chromatography–tandem mass spectrometry (HPLC-MS/MS). Under the condition of mass spectrometry, the electrospray source positive ion mode was used for the operation. The ion source temperature was set at 450°C and the spray voltage at 5,500 V. The MRM mode was used for data acquisition and detection. Based on the difference in molecular weight and mass charge ratio, HPLC-MS/MS could distinguish VD_2_ and VD_3_ (4).

### Data analysis

SPSS 21.0 software was used to analyze the data. Measurement data that conformed to normal distribution were expressed as mean ± standard deviation. The differences between groups were compared via *t*-test; if the data were non-normally distributed, the Mann–Whitney *U* test was used. *One-way ANOVA* test analysis was used to compare the VD_2_ levels of children of different ages. The difference was considered as statistically significance with *P *< 0.05.

### Role of the funding source

The funding agencies did not participate in the study design, data collection, data analysis, or manuscript writing. The corresponding authors were responsible for all aspects of the study to ensure that issues related to the accuracy or integrity of any part of the work were properly investigated and resolved. The final version was approved by all authors.

## Results

### 25(OH)D_2_ concentration in all age groups

The results presented in Table 1 demonstrated variations in the concentration of 25(OH)D_2_ among children across different age groups. The highest levels of 25(OH)D_2_ were observed in children aged 2–6 years, while a gradual decrease was observed in children aged 0–1 years, 7–10 years, and 11–15 years. Multiple comparisons revealed that the levels of 25(OH)D_2_ in children aged 7–10 years and 11–15 years were significantly lower than those in children aged 2–6 years (*P *= 0.042).

**Table 1:** Concentration of 25(OH)D_2_ in different age groups (ng/ml).

Age	Characteristics (*N*)	25(OH)D_2_	Standard deviation	95% CI
0–1	3,130	1.87	2.33	1.79–1.95
2–6	3,636	2.57	3.62	2.45–2.69
7–10	2,715	1.50	2.21	1.42–1.58
11–15	2,025	1.05	0.78	1.02–1.08

### 25(OH)D_2_ deficiency in different age groups

The deficiencies of 25(OH)D_2_ were severe in children across all age groups. As depicted in Table 2, the severity was highest among children aged 11–15 years, followed by those aged 7–10 years, 0–1 years, and 2–6 years. The proportion of individuals with a level of 25(OH)D_2_ lower than 1.5 ng/ml to the total population exhibited the highest value of 86.37%, lowest value of 68.34%, and average value of 78.61%.

**Table 2:** The 25(OH)D_2_ deficiencies in different age groups.

Age	Characteristics (*N*)	#25(OH)D_2_ <1.5 ng/ml/*N*
0–1	3,130	74.98%
2–6	3,636	68.34%
7–10	2,715	84.75%
11–15	2,025	86.37%

N, the number of people tested; # number.

### Effect of gender on 25(OH)D_2_ deficiency

This study analyzed the prevalence of 25(OH)D_2_ deficiency in boys and girls across different age groups. The results indicated that there was no significant difference in 25(OH)D_2_ levels between boys and girls (*P *= 0.28).

### 25(OH)D_2_ deficiency in departments with higher detection frequencies

By analyzing the data from the orthopedics, dermatology, thoracic surgery, and nephroimmunology departments, the findings revealed that the average proportion of individuals with 25(OH)D_2_ levels below 1.5 ng/ml to those with 25(OH)D levels between 15 and 100 ng/ml was 76.56%. Moreover, the average ratio of individuals with 25(OH)D_2_ levels less than 1.5 ng/ml to those with 25(OH)D levels lower than 15 ng/ml was found to be approximately 91.22%. The 25(OH)D_2_ deficiencies among children in each department are summarized in Table 3.

**Table 3:** The 25(OH)D_2_ deficiencies in four departments.

Department	Disease	*N*	#25(OH)D_2_ <1.5 ng/ml/#25(OH)D 15–100 ng/ml	#25(OH)D_2_ <1.5 ng/ml /#25(OH)D <15 ng/ml
Orthopedics	Osteomyelitis	115	74.74%	95.00%
	Abnormal gait	456	83.07%	89.47%
	Limb pain	901	78.14%	98.43%
	Congenital tibial curvature	158	76.16%	100.00%
	Congenital genu valgum	214	80.19%	100.00%
Dermatology	Areata	66	82.00%	100.00%
	Vitiligo	98	69.77%	100.00%
	Atopic dermatitis	44	90.24%	100.00%
	Eczema	84	91.36%	100.00%
	Urticaria	38	76.67%	87.50%
	Rash	144	85.61%	80.00%
	Dermatitis	81	86.30%	87.50%
Thoracic	Chest pain	65	79.07%	100.00%
	Funnel chest	166	69.80%	94.12%
	Rickets	75	10.81%	0.00%
	Chest wall deformity	446	74.41%	87.50%
Nephroimmunology	Systemic lupus erythematosus	43	79.41%	70.00%
	IgA nephropathy	13	80.00%	100.00%
	Allergic purpura	25	81.82%	100.00%
	Nephrotic syndrome	223	88.89%	92.31%

*N*, the number of people tested; # number.

## Discussion

As a fat-soluble vitamin, VD is an essential nutritional element for maintaining human health. Its primary functions include promoting the absorption of calcium and phosphorus by small intestinal mucosal cells, stimulating the growth and differentiation of skin cells, and regulating immunity. The hormone–receptor complex formed when 1,25-(OH)_2_-D binds to the VD receptor in the nucleus acts on specific DNA sequences within target genes, thereby influencing various aspects (5). Research has confirmed that many diseases have low levels of 25(OH)D in patients, such as diabetes (6), hypertension (7), cardiovascular disease (8), and cancer (9).

Although VD plays a crucial role in the human body, VD deficiency remains a global public health issue (10), particularly among children. Due to their rapid growth and high nutrient demands, such as VD, children are more susceptible to nutritional deficiencies. Studies conducted in various countries have reported a high prevalence of VD deficiency across different age groups, similar to other populations worldwide (11, 12). However, many of these studies failed to differentiate VD types and defaulted to considering only VD_3_ while disregarding the presence of VD_2_. Specifically, the side chain of VD_2_ contains a double bond between carbons 22 and 23, with a methyl group attached at carbon 24, whereas VD_3_ lacks both groups. Moreover, VD_2_ also has the characteristic of fast metabolism, which may be related to the low level of VD_2_ in the body. Although it exists in low quantities among humans, VD_2_ plays an essential physiological role. Yang found that combining Shenqi Jianer decoction with VD_2_ injection effectively improved serum calcium and phosphorus levels while reducing bone alkaline phosphatase levels in children with rickets (13). Su et al. found that intramuscular injection of VD_2_ significantly improved the status of VD deficiency in patients with IgA nephropathy and reduced proteinuria (14). Li et al. found that high-dose intramuscular administration of VD_2_ was beneficial for type 2 diabetic patients with distal symmetric polyneuropathy (15).

In this study, HPLC-MS/MS was utilized for assessing 25(OH)D and 25(OH)D_2_ levels due to its high sensitivity and specificity, providing certain methodological advantages (16).The investigation aimed to analyze the levels of VD_2_ in pediatric patients attending the hospital. This study found that there were differences in the levels of VD_2_ among children of different age groups, but there was no significant difference in VD_2_ levels between boys and girls. The reasons for the differences in the degree of VD_2_ deficiency among children of different age groups may include different individual needs for VD_2_, different exposure times to sunlight, and different levels of VD_2_ intake. VD_2_ can only be obtained from external sources. Notably, Simon et al. found that mushrooms are good sources of VD_2_ (17). Mushrooms contain ergosterol, a precursor that enables them to synthesize VD_2_ when exposed to sunlight or ultraviolet light. Bisporus mushrooms are rich in high nutritional value and have numerous therapeutic benefits. Therefore, they are widely used in countries around the world.

In addition, by analyzing the VD_2_ levels of children in departments with a high frequency of VD detection, the findings revealed that not only children with orthopedic and thoracic surgery-related diseases exhibited VD_2_ deficiency but also those with non-skeletal-related conditions such as skin and kidney diseases. Further data analysis indicated that 76.56% of the children had VD_2_ deficiency despite their VD values falling within the normal range. Moreover, among children with severely deficient VD levels, 89.19% exhibited insufficient VD_2_ levels. For specific conditions such as congenital tibial bending and curvature of the tibia in orthopedics, areata, vitiligo, atopic dermatitis, and eczema in dermatology, chest pain in thoracic surgery, and IgA nephropathy and allergic purpura in nephroimmunology, if there were VD deficiencies, these children would have 100% rates of VD_2_ deficiency. Another study suggested that populations undergoing VD_2_ testing were 4.2 times more likely to check out VD deficiency compared to those without VD_2_ testing (18). In conclusion, assessing the level of VD_2_ was highly significant for evaluating an individual's VD status.

Therefore, it was recommended that the detection of VD_2_ should be conducted simultaneously with VD detection in order to more accurately assess the nutritional status of children. Furthermore, considering a limitation of this study was that samples from hospitals were utilized, the generalizability of the findings may be impacted. Additionally, other factors such as seasonal variations, daily dietary intake, and individual sun protection behavior that could potentially affect VD content need to be further taken into account. Hence, more comprehensive research is warranted to investigate the VD_2_ deficiency and the associations between VD_2_ and specific diseases in order to provide better guidance for clinical practice.

## Data Availability

The datasets presented in this article are not readily available because patient privacy concerns. Requests to access the datasets should be directed to ljbrave1@163.com.
